# Supplementary material for: Physiological Demands of a Self-Paced Firefighter Air-Management Course and Determination of Work Efficiency
Source: J Funct Morphol Kinesiol. 2023 Feb 6;8(1):21. doi: 10.3390/jfmk8010021 (PMC9944468; doi:10.3390/jfmk8010021)
Supplement: Supplementary file 1 [file jfmk-08-00021-s001.zip › jfmk-2178170-supplementary.pdf]

Supplementary figures

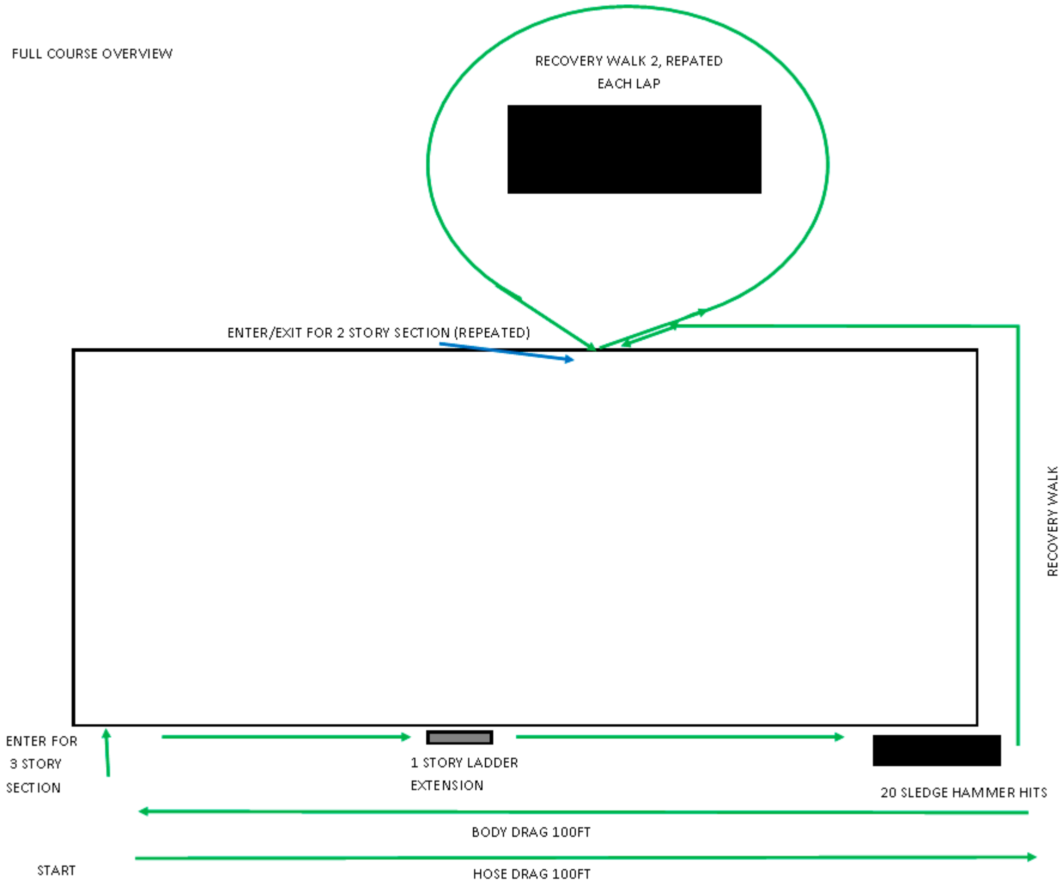

Figure S1: SF1 Air Management Course Schematic, Full Course Overview

SECOND FLOOR OVERVIEW

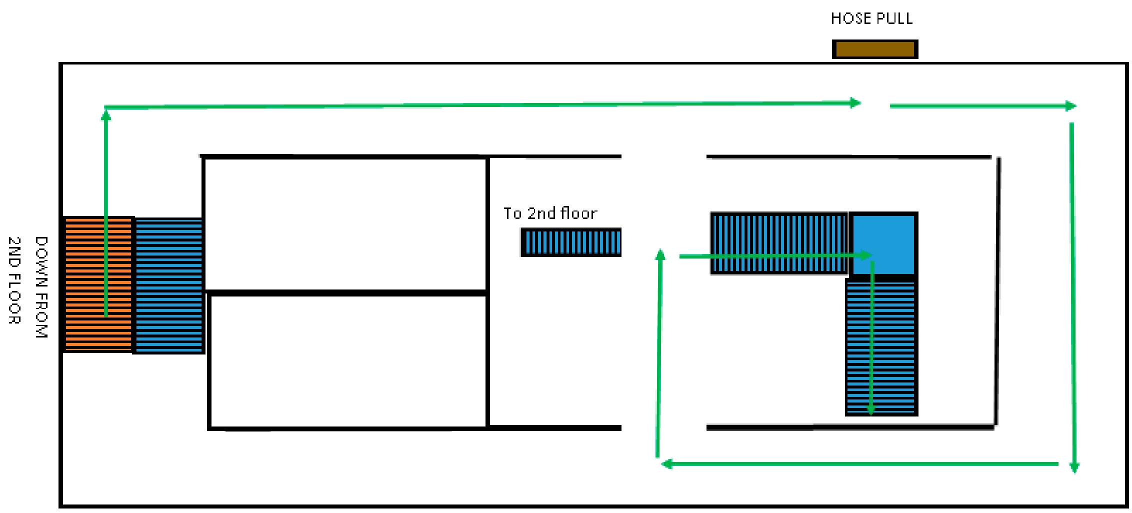

Figure S2: SF2 Air Management Course Schematic, Second Floor Overview

THIRD FLOOR OVERVIEW

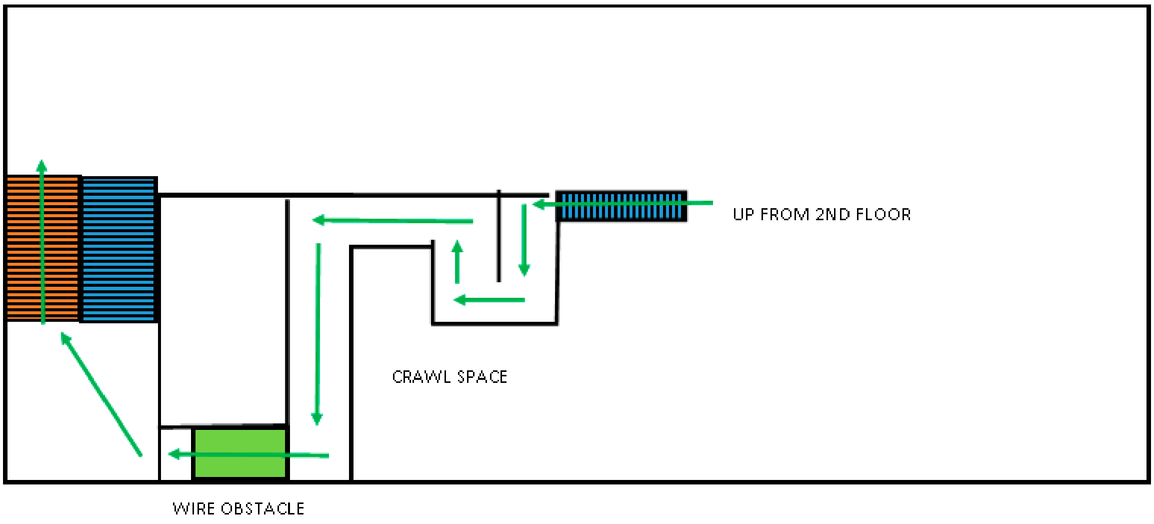

Figure S3: SF3 Air Management Course Schematic, Third Floor Overview

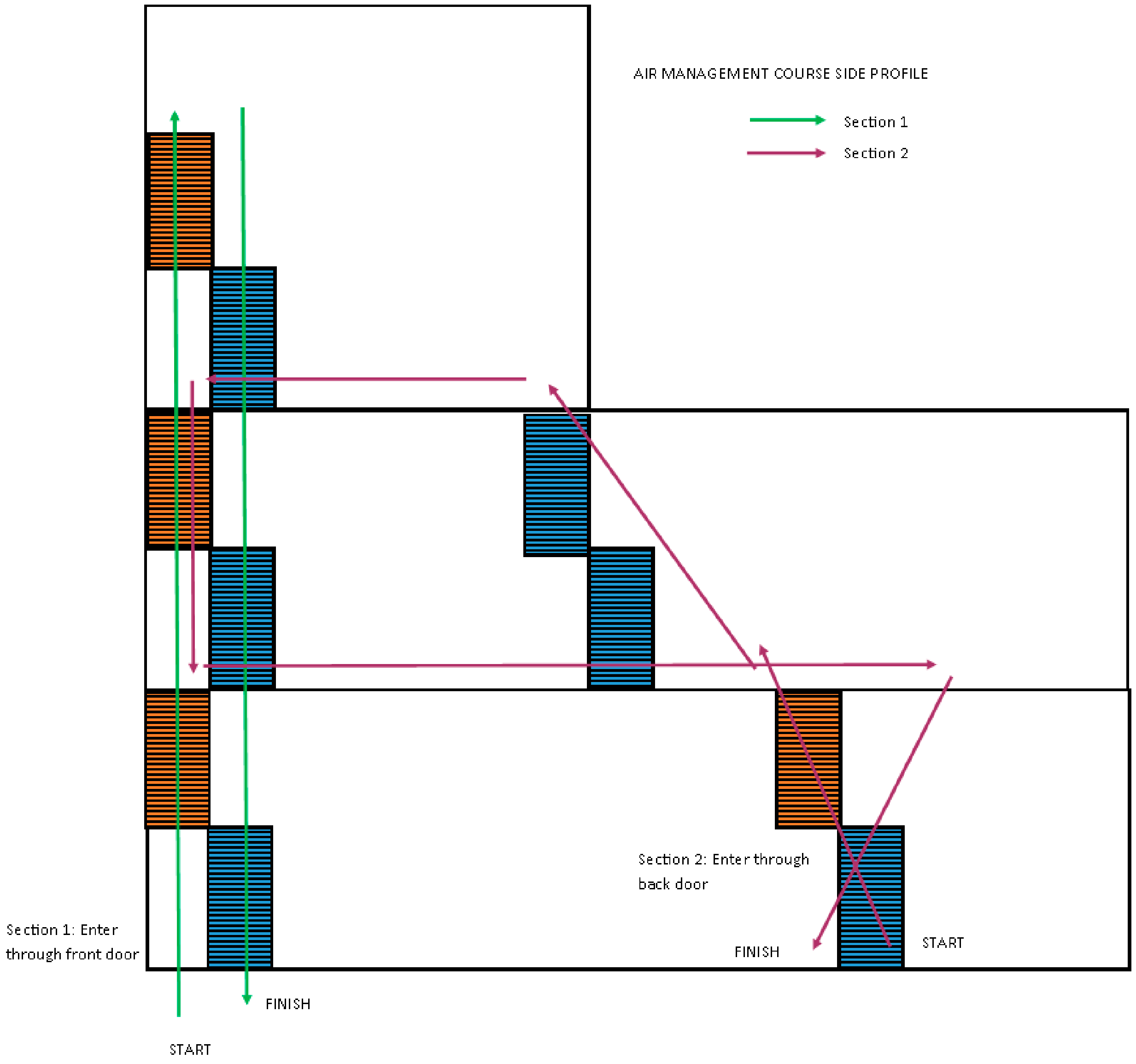

Figure S4: SF4 Air Management Course Schematic, Side Profile View

Description

These figures represent the layout and direction of movement for the air management course.
